# A qPCR method to quantify bioavailable phosphorus using indigenous aquatic species

**DOI:** 10.1186/s12302-018-0163-z

**Published:** 2018-09-04

**Authors:** Yanan Yang, Jianghua Yang, Xiaowei Zhang

**Affiliations:** State Key Laboratory of Pollution Control & Resource Reuse, School of the Environment, Nanjing University, 163 Xianlin Avenue, Nanjing, 210023 China

**Keywords:** Cyanobacteria, Algae bloom, Eutrophication, Bioreporters, Alkaline phosphatase, Phosphate transporter genes

## Abstract

**Background:**

Bioavailable phosphorus (BAP) represents the sum of phosphorus that is readily available for algae growth and is useful to indicate the severity of eutrophication in aquatic environments.

**Results:**

Here, a quantitative real-time PCR (qPCR)-based bioassay was developed to quantify BAP using the indigenous cyanobacterium species *Anabaena* sp. of Lake Tai, a large and shallow eutrophic lake in the Yangtze Valley, China. Primers were designed to quantify the gene expression of alkaline phosphatase (*phoA*/*phoA*-*like)* and phosphate transporter (*pst1*) genes of *Anabaena*. The specificity and efficiency of the primer sets were evaluated by gel electrophoresis and real-time PCR. The results showed that the primers developed here could successfully be used to measure BAP in the water. The linear range of BAP measurements by the *pst1* gene after 2 h incubation was 0.125–2.00 mg/L. Then, the qPCR-based bioassay was applied to analyze water samples from Tai Lake, which had BAP levels in the range of 0.239–0.459 mg/L.

**Conclusions:**

The qPCR-based bioassay represents a promising biomonitoring tool that can quantify phosphorus bioavailability in aquatic environments.

## Background

The eutrophication of aquatic ecosystems is a major environmental issue threatening water security and biodiversity. In recent years, lake eutrophication has intensified globally due to human activities such as aquaculture, agricultural fertilization, sewage discharge and tourism. Eutrophication causes algal blooms, hypoxia, acidification and fish deaths, raised water purification costs, and results in the loss of economic benefits associated with clean water [1]. Phosphorus is an important nutrient that restricts microbial production in freshwater and marine environments [2, 3]. A steady increase in phosphorus loading in a lake is usually the most important cause of eutrophication, causing rapid increase in algal productivity when the biological productivity of the lake is low or intermediate [4].

Phosphorus in water can be found in different organic or inorganic and dissolved or particulate forms. However, not all forms of phosphorus are equally important for eutrophication. Bioavailable phosphorus (BAP) is defined as the sum of immediately available phosphorus, which can be transformed into an available form by naturally occurring processes [5]. BAP is closely related to the growth of aquatic organisms. Therefore, measuring BAP in water is important to indicate the severity of eutrophication and provide early warnings for algal bloom outbreaks.

While analytical technologies measure a limited number of phosphorus species, bioassays can be used to detect all forms of BAP and indicate their biological effects [6]. Usually, algae cultures are used to estimate the bioavailable phosphorus content in water and sediment. Phosphatase activity is often a good indicator of phosphorus limitation [7]. Cyanobacteria respond to P-limiting conditions by increasing the surface phosphatase activities (such as alkaline phosphatases), and phosphatase activities will be completely suppressed under high ambient phosphate concentrations [7]. Phosphate-limited cyanobacteria will also increase the phosphate uptake rates [8]. *Anabaena* is a representative cyanobacteria, which are the most ancient phytoplankton on the planet and cause harmful algal blooms. Several model bacteria have been found to respond genetically to P limitation by up-regulating the expression of a series of genes, such as *pho* genes encoding alkaline phosphatases (APases) or *pst* genes encoding phosphate transporters that constitute a Pho regulon [9, 10]. The enzymes APases play a crucial role in the metabolism and regulation of phosphorus because they can catalyse the non-specific hydrolysis of phosphoesters or phosphodiesters to produce Pi [11].

**Fig. 1:**
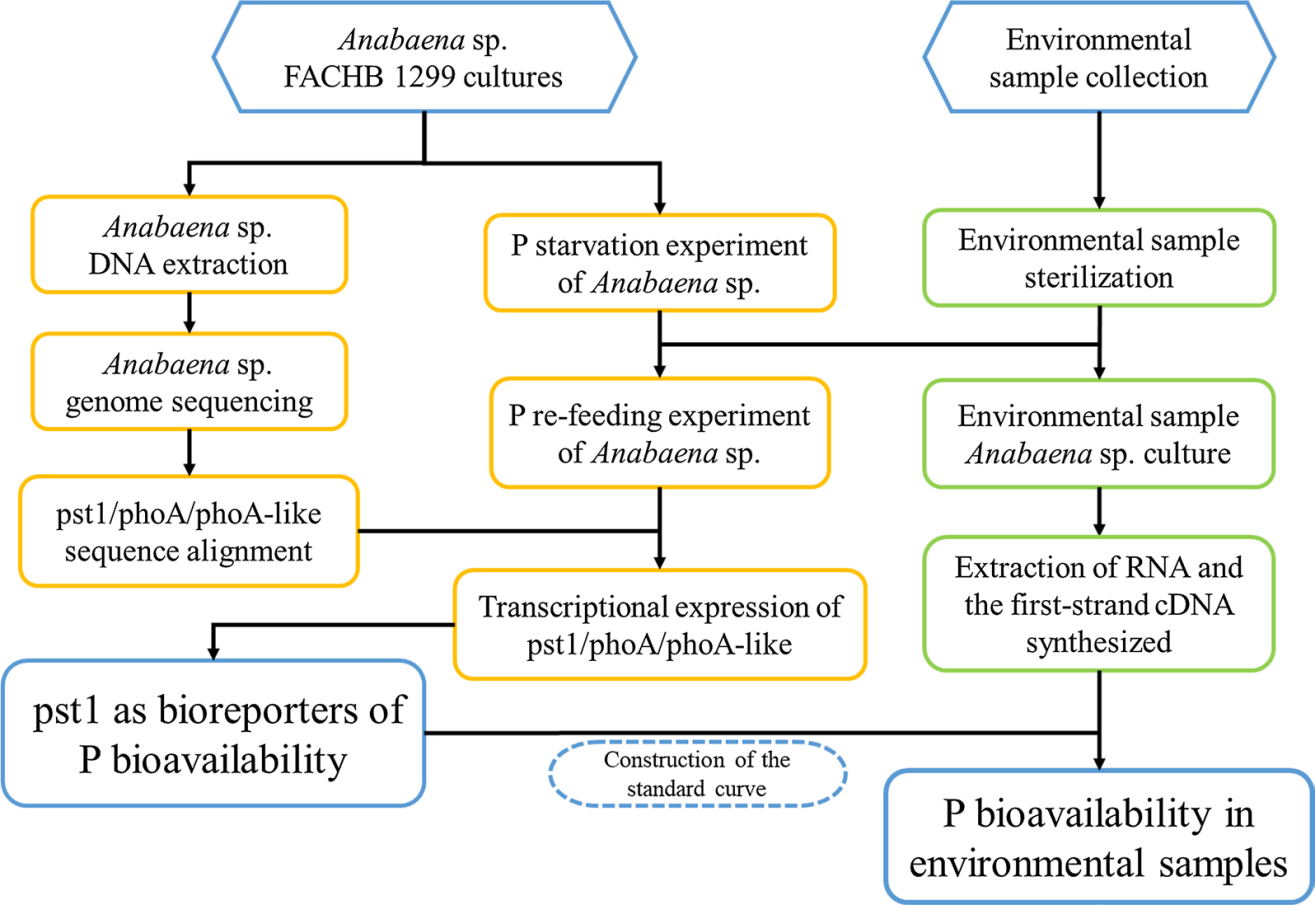
Technology roadmap

The catchment of Lake Tai is one of the most densely populated and developed areas in China. But excessive nutrient loading by rapid industrialization and urbanization has caused rapid deterioration of water quality. Indigenous species are useful tools to provide ecologically relevant bioindicators of environmental change. Here, a quantitative real-time PCR (qPCR)-based bioassay was developed to assess phosphorus bioavailability in the water by measuring the transcriptional activity of alkaline phosphatase and phosphate transporter genes in the indigenous cyanobacterium species *Anabaena* sp. FACHB-1299 (Fig. 1).

## Methods

### Bacterial strains and culture conditions

The cyanobacterium *Anabaena* sp. FACHB 1299 was obtained from the Freshwater Algae Culture Collection of the Institute of Hydrobiology (FACHB-Collection; Wuhan, China). FACHB 1299 and its derivatives were cultured in BG-11 medium at 28 °C [12]. In P-depleted medium, K_2_HPO_4_ was replaced by equimolar amounts of KCl.

### *Anabaena* sp. strain FACHB-1299 genome sequencing

Because the genomic sequence of FACHB-1299 was not available, an analysis of the genome sequence of *Anabaena* sp. strain FACHB-1299 was first conducted. Total DNA was extracted from the *Anabaena* sp. FACHB 1299 culture and subjected to sequencing by the Personal Genome Machine (PGM) (Life Technologies, CA, USA). Bioinformatics analysis revealed that the genome of *Anabaena* sp. FACHB-1299 is approximately 11.3 Mbp in size. The complete annotation of the full genome is in progress. The DNA sequence alignment method was adopted for the full-length sequences of alkaline phosphatase and phosphate transporter genes.

### Environmental water sampling in Lake Tai

Lake Tai is a large and shallow eutrophicated lake in the Yangtze valley. Surface water (30 cm under water) was sampled from 7 sampling sites (Table 1) across Lake Tai by a vertical water extractor from April 16 to 20, 2015. For each sampling site, 250 mL of the water sample was filtered immediately after sampling with a 0.22 μm Millipore membrane (Millipore), and the water was stored at 4 °C until use.

**Table 1:** Geographical features of sampling sites

Sample ID	Sample name	Longitude	Latitude
01	Xishanxi	120.150	31.140
02	Zeshan	120.268	31.014
03	Dongtaihu	120.507	31.071
04	Puzhuang	120.453	31.186
05	Jinshugang	120.361	31.384
06	Wuguishan	120.229	31.310
07	Tuoshan	120.162	31.392

### P starvation and P re-feeding experiment

The cultures were pre-grown for 3 days in complete medium starting at an optical density of 0.2 at 750 nm (OD750). *Anabaena* sp. cells were collected from the mid-logarithmic-phase cultures by centrifugation and washed twice with P-depleted BG-11 medium. The cells were subsequently inoculated into P-depleted BG-11 for further growth. For the P re-feeding experiments, cells grown for 72 h in P-depleted medium were harvested by centrifugation at 3000×*g* with 30 min. An aliquot of 20 mL cells was added to 180 mL of P-depleted medium supplemented with different concentrations of K_2_HPO_4_. Cells were harvested after 2 h, 4 h and 8 h by centrifugation for 10 min at 23,000×*g* for RNA isolation.

### Extraction of RNA and first-strand cDNA

*Anabaena* sp. RNA was extracted with the RNeasy Plant Mini Kit (QIAGEN, Germany), and RNA was determined by Qubit (Thermo Fisher, USA). First-strand cDNA was synthesized from total RNA with ReverTra Ace qPCR RT Kit (Toyobo, Shanghai, China) in accordance with the manufacturer’s instructions.

### Real-time PCR analysis

Three genes encoding alkaline phosphatases and phosphate transporters were selected from the *Anabaena* sp. FACHB-1299 genome. The primers used for amplifying each gene were designed using Primer 5.0 (Primer, Canada) (Table 2). To verify that each primer hybridized to the target sequence only, gradient PCR were performed before the quantitative PCR. To determine the amplicon identity, all of the PCR products were cloned into PMD19-T vectors, and sequenced at Generay Co. (Shanghai, China). PCR product was analyzed on a 0.2% agarose gel. QPCR amplification and analysis were performed using the StepOne Real-Time PCR Systems (Thermo Fisher, USA). All reactions were performed using the StepOne Real-Time PCR Systems (Thermo Fisher, USA) according to the manufacturer’s instructions. The PCR reaction conditions were as follows: pre-incubation at 95 °C for 10 min; 40 cycles at 94 °C for 10 s, 60 °C for 30 s, and 72 °C for 30 s and a final extension at 72 °C for 3 min. Fluorescence was measured at the end of each annealing step. Amplification was followed by a melting curve analysis with continual fluorescence data acquisition during the 56–61 °C melt. The raw data were analysed with the StepOne Real-Time PCR Systems (Thermo Fisher, USA), and the gene expression levels were normalized to *Anabaena* sp. *16S* (accession number 14088448) to minimize variations in the cDNA template. QPCR data were technical replicates with error bars, representing mean ± SE (*n *= 3). Statistical and correlation analyses were performed with SPSS. The 2^−ΔΔCt^ method [13] was used to calculate the relative expression of the phosphorus metabolism-related enzyme genes.

**Table 2:** Primers used to quantify the transcriptional activity of alkaline phosphatase and phosphate transporter genes in *Anabaena* sp. FACHB 1299

Primer	Gene accession	Sequence (5′–3′)	Tm (°C)	Product (bp)
pst1-F	MH184530	GCCACAGCTCAAGCTCAAAC	60	138
pst1-R		CCCACCACCACTACCAATCC		
phoA-F	MH184531	GTGGCTGGAGCAAGAACTTA	60	171
phoA-R		CAGCATCTTGAGGGTTGTGT		
phoAlike-F1	MH184532	TCGGCAGGAATAGTCAAGGT	60	124
phoAlike-R1		AAGTCATCGCCACTGTCGTA		
16s-F	14088448	AAGCATCGGCTAACTCC	60	199
16s-R		TTTCACCGCTACACCAG		

## Results and discussion

### Transcriptional response of *phoA*-*like*, *pst1*, and *phoA* to phosphate

**Fig. 2:**
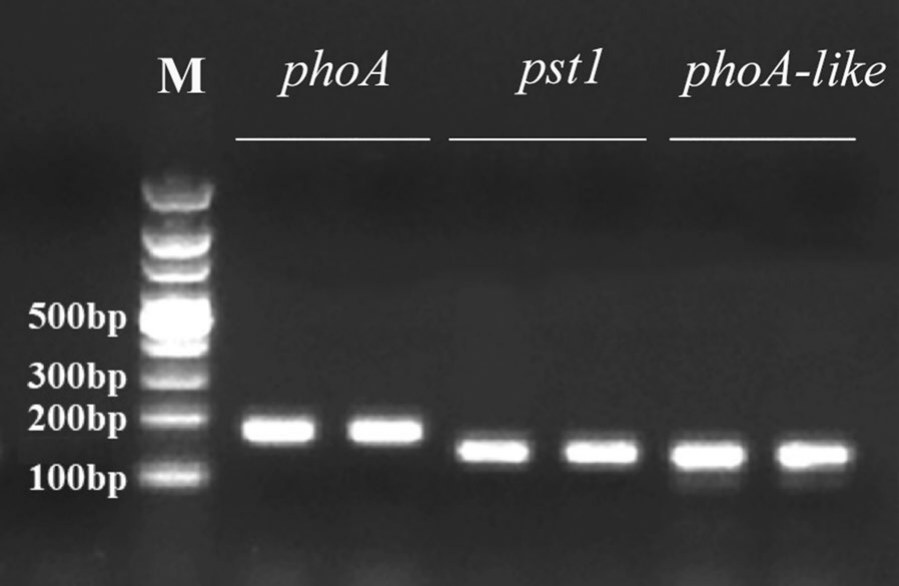
Gel electrophoresis of the PCR products by the designed primers using the genomic DNA template of cyanobacterium *Anabaena* sp. FACHB 1299

The targeted cDNA of alkaline phosphatase genes in FACHB 1299 was successfully detected by the three primer sets (Fig. 2). PCR with the pst1F/pst1R primer set produced an amplicon with a size of approximately 138 bp. The results of cloning and sequencing confirmed the PCR products by the three primer sets, pst1F/pst1R, phoA-F/R and phoAlike-F1/R1, with complete matches to the corresponding genome sequence.

**Fig. 3:**
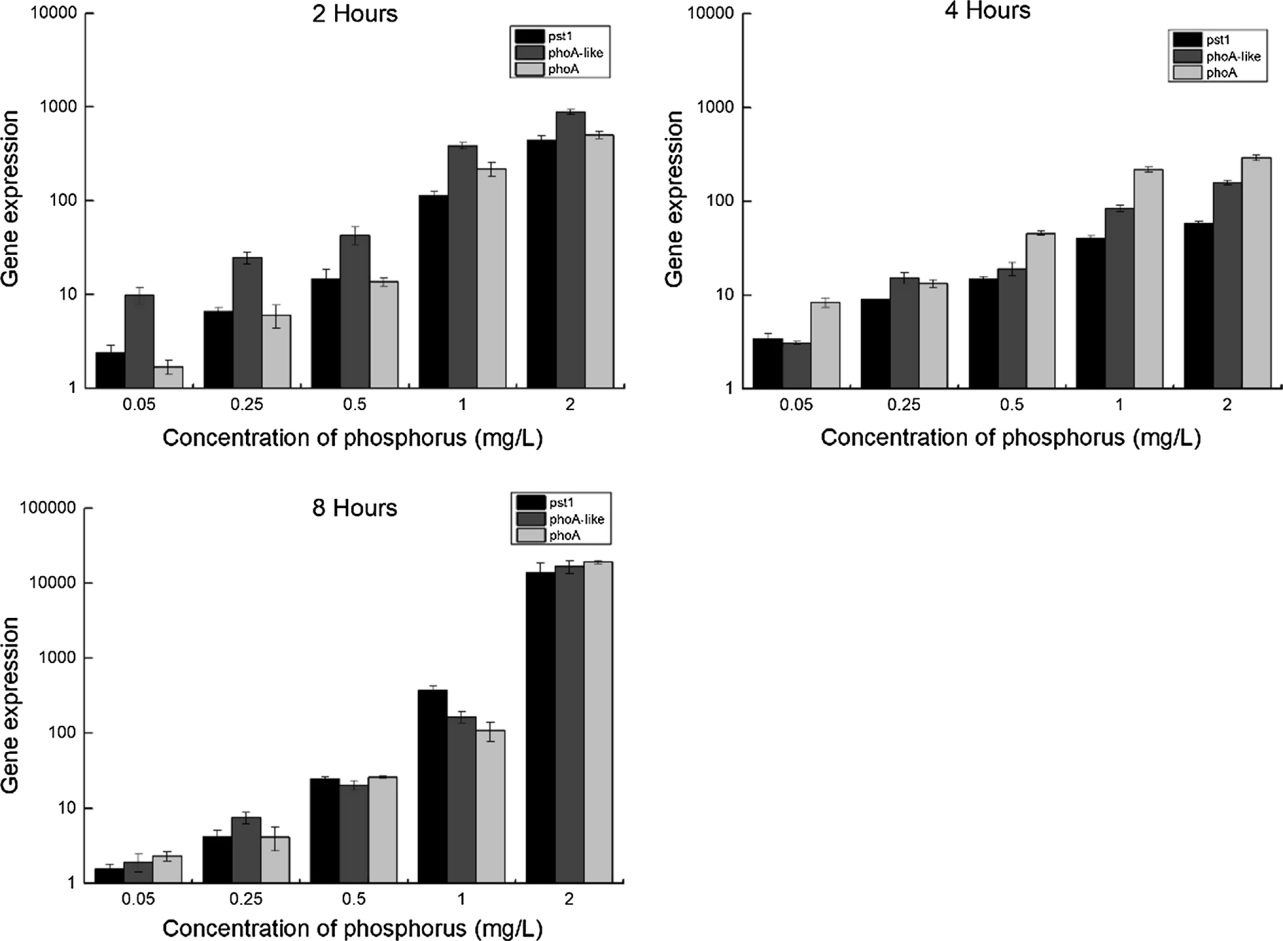
Gene expression of the three studied genes (*phoA*/*phoA*-*like*/*pst1*) in response to phosphorus (K_2_HPO_4_) exposure after 2, 4, and 8 h of incubation

A concentration-dependent increase in transcriptional expression was observed for each of the three genes at 2 h, 4 h or 8 h after P re-feeding (Fig. 3). These patterns were consistent with previous observations made in another *Anabaena* sp. stain, PCC 7120, using a fluorescent reporter gene approach [14]). Another study in *Anabaena* sp. FACHB 709 showed four APases (phoA-709, phoD1-709, phoD2-709, and phoS-709) were involved in P metabolism and regulation, and PhoA-709 was the main APase involved in these processes [15].

**Fig. 4:**
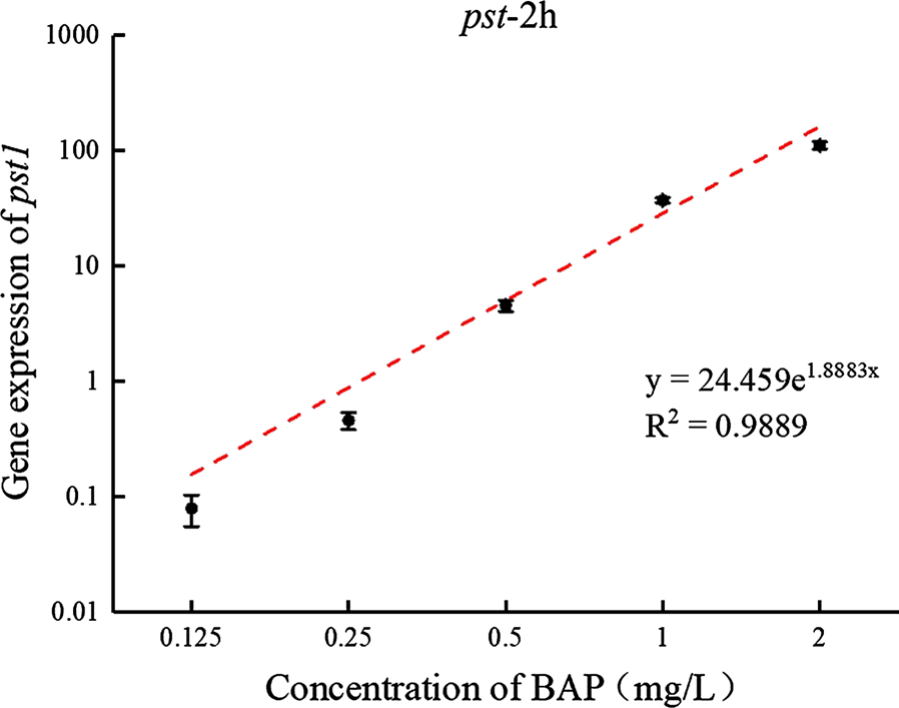
Standard curve based on the mRNA expression of *pst1*-2 h in different concentrations of phosphorus (K_2_HPO_4_)

It has been previously shown that *pst1* is activated at a much higher level than *phoA*-*like* and *phoA* following P starvation [14].And the 2 h is more convenient than 4 h and 8 h for the operators. Therefore, the expression level of pst1 at 2 h was used as a bioindicator for BAP. A linear increase in *pst1* gene expression at 2 h was observed in the full concentration range, which can be used as the standard curve for P bioavailability quantification (Fig. 4).

### pst1 as bioreporters of P bioavailability in environmental samples

The BAP contents in the water from Lake Tai ranged from 0.239 to 0.459 mg/L based on the expression trend of *pst1*-2 h in the QPCR bioassay (Table 3). Although this is just one-time measurement, the result showed that this value was within the total P (TP) content range (0.051–0.770 mg/L) of Meiliang Bay of Tai Lake in 2013. A previous BAP measurement by the algae culture method provided a lower BAP concentration range (0.023–0.107 mg/L) [16], this difference might be due to differences in the technologies used. Together with the methods of this study, the biological-based approach can be supplementary to TP content monitoring in freshwater management.

**Table 3:** Concentrations of bioavailable phosphorus (BAP) in different sampling sites

Sample ID	Sample sites	Relative gene expressions	BAP (mg/L)
01	Xishanxi	2.163	0.411
02	Zeshan	1.979	0.397
03	Dongtaihu	0.538	0.246
04	Puzhuang	2.027	0.401
05	Jinshugang	0.495	0.239
06	Wuguishan	2.389	0.426
07	Tuoshan	2.936	0.459

The qPCR-based bioassay represents a promising biomonitoring tool that can quantify phosphorus bioavailability in aquatic environments. Many methods have been used to measure the dissolved inorganic phosphorus fraction, including electrochemical, chromatographic and enzymatic assays [17]. However, most of these chemical approaches may not be able to estimate the actual bioavailable phosphorus because of a lack of sensitivity and inability to be applied to environmental samples. An inverse relationship was found between values of bioavailable P, measured by enzymatic assays and phosphatase activities. Cyanobacteria from sampling sites with low bioavailable P showed high phosphatase activity and vice versa [18]. The standard algal available P (AAP) test provides biological estimates of bioavailable and limiting nutrients via extensive evaluations and applications [19, 20]. The BAP in sediments from West Lake and Lake Tai (China) and Lough Erne (Northern Ireland) has been evaluated using total P (TP), water soluble P (WSP), readily desorbable P (RDP), algal available P (AAP) and Olsen-P. The rank order of the extraction efficiency was the same in all lakes in the sequence and was as follows: AAP > Olsen-P > WSP > RDP [21]. The molecular method can be more sensitive and precise than the above methods for the detection of bioactive phosphorus. Quantitative RT-PCR from cultured marine *Synechococcus* sp. strain WH8102 and freshwater *Synechococcus* sp. ARC-21 demonstrated the induction of phnD expression in P-depleted media, suggesting that *phn* genes are regulated coordinately with genes under phoRB control [18].

## Conclusion

In summary, a qPCR bioassay was developed to quantify phosphorus bioavailability in aquatic environments. The results showed that the primers designed in this study could successfully detect phosphorus bioavailability in the water. Overall, these bioreporters provide information on the BAP or of a specific analyze to the indigenous species. Future technological developments may make this method much more available for standardized application for environmental studies.

## Abbreviations

BAP: bioavailable phosphorus
qPCR: quantitative real-time PCR
APases: alkaline phosphatases
FACHB-collection: the Freshwater Algae Culture Collection of the Institute of Hydrobiology
TP: total phosphorus
AAP: algal available phosphorus
WSP: water soluble phosphorus
RDP: readily desorbable phosphorus
